# Therapeutic Effect of Membrane Vesicle Drug Delivery Systems in Inflammatory Bowel Disease

**DOI:** 10.3390/pharmaceutics17091127

**Published:** 2025-08-28

**Authors:** Zhe Zhao, Ziyun Li, Yihuang Gu, Renjun Gu

**Affiliations:** 1 Department of Gastroenterology and Hepatology, Jinling Hospital, Affiliated Hospital of Nanjing University of Chinese Medicine, Nanjing 210000, China; zz1535962@163.com; 2 School of Acupuncture and Tuina, School of Regimen and Rehabilitation, Nanjing University of Chinese Medicine, Nanjing 210023, China; ziyunli@njucm.edu.cn; 3 School of Chinese Medicine, Nanjing University of Chinese Medicine, Nanjing 210023, China

**Keywords:** membrane vesicle, drug delivery systems, inflammatory bowel disease, mechanisms

## Abstract

Inflammatory bowel disease (IBD) is a chronic, heterogeneous condition characterized by recurrent intestinal inflammation and sustained mucosal barrier damage, profoundly impairing patients’ quality of life and imposing a considerable socioeconomic burden. Current therapeutic options are often constrained by low oral bioavailability, pronounced systemic toxicity, and inadequate tissue specificity, limiting their ability to achieve precise and durable efficacy. In recent years, membrane vesicle-based drug delivery systems (MV-DDSs) have shown considerable promise for precision IBD therapy owing to their excellent biocompatibility, mucosal barrier-penetrating capacity, and low immunogenicity. Building upon a systematic discussion of the roles of MV-DDSs in suppressing inflammatory signaling, modulating oxidative stress, preserving barrier integrity, reshaping the gut microbiota, and regulating programmed cell death, this review further compares the differences in key molecular targets and functional outcomes among vesicles of diverse origins and carrying distinct therapeutic payloads. These insights provide a comprehensive strategic reference and theoretical foundation for the rational design, mechanistic optimization, and clinical translation of MV-DDSs in IBD therapy.

## 1. Introduction

Inflammatory bowel disease (IBD) is a chronic, recurrent inflammatory disease that primarily affects the digestive system. The main subtypes of IBD are ulcerative colitis (UC) and Crohn’s disease (CD) [1]. The exact mechanisms underlying this disease have not yet been fully elucidated, but increasing evidence suggests that IBD is closely associated with abnormal immune responses, intestinal barrier dysfunction, intestinal microbiota imbalance, and complex interactions between genetic predisposition and environmental factors [2,3]. IBD affects approximately 6 million people worldwide each year [4], imposing a substantial healthcare and socioeconomic burden, increasing the risk of colorectal cancer, and severely impairing patients’ quality of life [5].

In the pathogenesis of IBD, abnormal activation of the immune system causes a persistent and excessive immune response against the host’s own intestinal tissue. This dysregulated immune response is associated with excessive production of proinflammatory cytokines and accumulation of reactive oxygen species (ROS), leading to complex amplification effects in inflammatory signaling [6]. At the same time, dysbiosis of the intestinal microbiota and dysfunction of the intestinal epithelial barrier further exacerbate the inflammatory environment, creating a vicious cycle of “barrier disruption–amplification of inflammation–progressive barrier impairment” [7]. Current standard treatment for IBD primarily involves 5-aminosalicylic acid (5-ASA) preparations, corticosteroids, immunosuppressants, and biological agents targeting inflammatory mediators [8,9]. Although these strategies are effective in the treatment of IBD to a certain extent, they still have inherent limitations, including a significant decline in curative effect in severe cases, obvious systemic toxicity, easy drug resistance, insufficient tissue specificity, and difficulty in maintaining stable therapeutic drug concentrations in inflammatory sites [10]. Consequently, repeated or intensified dosing is often required, compounding adverse effects and undermining long-term disease control [11,12].

Given the significant limitations of conventional pharmacotherapy in terms of therapeutic stability and safety, the development of drug delivery systems (DDSs) has emerged as a critical strategy to enhance the precision and effectiveness of IBD treatment [13]. In recent years, rapid advances in nanotechnology have driven the innovation of various delivery platforms. Among these, membrane vesicles (MVs) have attracted increasing attention as novel drug delivery vehicles. Owing to their unique closed bilayer architecture and programmable surface functionalization, MVs are capable of optimizing multiple aspects of drug performance, including improved physicochemical stability, prolonged systemic circulation, enhanced targeting efficiency, and controlled release profiles [14].

**Figure 1:**
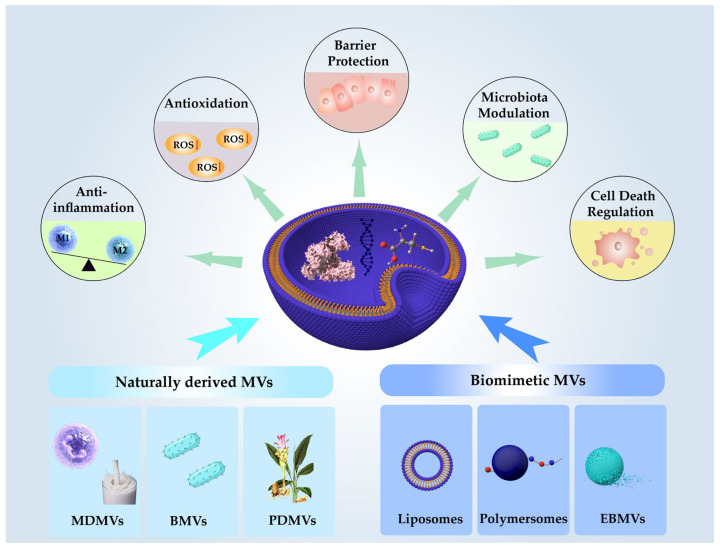
Origin of MV-DDSs and therapeutic mechanism in IBD. MV-DDSs can be classified according to their origin into mammalian-derived membrane vesicles (MDMVs), plant-derived membrane vesicles (PDMVs), bacterial membrane vesicles (BMVs), as well as liposomes, polymersomes, and engineered biomimetic membrane vesicles (EBMVs). These vesicles are capable of encapsulating a wide range of therapeutic cargos, including proteins, nucleic acids, and small-molecule drugs. In IBD therapy, MV-DDSs exert their effects through multiple mechanisms, such as suppressing inflammatory responses, scavenging ROS to alleviate oxidative stress, protecting intestinal barrier integrity, modulating gut microbiota composition, and regulating programmed cell death pathways.

Although numerous studies have explored the drug delivery strategies and therapeutic potential of MVs derived from various sources in diseases such as cancer and atherosclerosis [15,16], there remains a lack of systematic and integrative evaluation regarding their mechanistic effects in IBD. To address this gap, this review focuses on the functional mechanisms of MV-DDSs in the treatment of IBD. Specifically, we systematically review the therapeutic mechanisms of MV-DDSs in several key pathological dimensions, including the regulation of inflammatory responses, attenuation of oxidative stress, restoration of intestinal barrier integrity, modulation of gut microbiota composition, and intervention in programmed cell death pathways. By analyzing MVs from diverse origins and their bioactive cargos, this work discovers key regulatory molecules with pivotal roles in modulation of the disease. Furthermore, we summarize current engineering strategies enhancing the targeting specificity and bioavailability of MVs (Figure 1). This review offers mechanistic insights and strategic guidance for the rational design of next-generation MV-DDSs tailored for precision therapy in IBD.

## 2. MVs

### 2.1. Overview of MVs

MVs are closed, near-spherical structures assembled from lipid bilayers or amphiphilic molecules, exhibiting membrane compositions and topological configurations that closely resemble those of natural biological membranes. This structural arrangement creates a relatively stable microenvironment within the vesicular lumen, capable of encapsulating a broad range of therapeutic cargos, including small-molecule drugs, proteins, and microRNAs, thereby markedly enhancing their physicochemical stability [17,18]. The lipid bilayer effectively shields the encapsulated agents from enzymatic degradation, pH fluctuations, and oxidative stress, prolonging their systemic half-life and preventing premature release before reaching the target site. This feature reduces non-specific toxicity to healthy tissues and ensures precise therapeutic action [19].

In terms of targeted delivery, MVs possess inherent targeting capabilities, as their surfaces are enriched with transmembrane proteins, glycoproteins, and adhesion molecules that can bind selectively to specific receptors on target cells, thereby facilitating active accumulation and uptake in desired tissues [20]. Furthermore, genetic engineering, chemical conjugation, or physical coupling strategies can be employed to graft targeting ligands (such as antibodies, peptides, or aptamers) onto the vesicle surface, further enhancing their selectivity and precision in delivering therapeutics to specific tissues or cells. These combined properties provide a robust platform for the targeted treatment of a wide spectrum of diseases.

### 2.2. Classification of MVs

Currently, MV-DSSs can be broadly categorized by their origin into two primary types: naturally derived MVs secreted by cells and biomimetic MVs synthesized through chemical or biophysical methods. Natural MVs can be further subdivided into three main classes based on their source: mammalian-derived membrane vesicles (MDMVs), plant-derived membrane vesicles (PDMVs), and bacterial membrane vesicles (BMVs). In contrast, artificial MVs are typically classified according to their fabrication strategies into liposomes, polymersomes, and engineered biomimetic membrane vesicles (EBMVs). Detailed information is provided in Table 1.

#### 2.2.1. MDMVs

MDMVs have demonstrated significant therapeutic potential in the treatment of IBD, owing to their natural origin, excellent biocompatibility, and complex functional cargo. Relevant examples are summarized in Table 2. Current research has primarily focused on MDMVs derived from mesenchymal stem cells (MSCs), immune cells, and mammary gland epithelial cells. Notably, vesicles secreted by different cellular sources exhibit distinct cargo profiles, targeting capabilities, and biological functions, which collectively determine their therapeutic applicability and efficacy in specific disease contexts.

Membrane vesicles derived from MSCs (MSC-MVs) have demonstrated promising therapeutic efficacy in experimental models of IBD. While MSC-MVs from different tissue sources share common capabilities in modulating immune responses and promoting tissue repair [34,35], their biological origin confers distinct functional characteristics. For instance, bone marrow-derived MSC-MVs (BM-MSC-MVs) are widely regarded as the benchmark in regenerative medicine, due to their potent immunomodulatory and regenerative properties. These vesicles are enriched in miRNAs such as miR-21, miR-125a/b, miR-146, and miR-181, which regulate key inflammatory pathways, including TGF-β, STAT3, and NF-κB [36,37,38]. Through these mechanisms, BM-MSC-MVs facilitate M2 macrophage polarization, inhibit Th17 cell differentiation, enhance intestinal epithelial barrier integrity, and mitigate inflammatory responses [37,39]. However, their isolation involves invasive procedures, limiting clinical scalability. In contrast, adipose tissue-derived MSC-MVs (AT-MSC-MVs) are more readily obtainable and exhibit a higher proliferative potential, along with a stronger capacity to secrete anti-inflammatory cytokines [40,41,42]. In addition to suppressing classic proinflammatory mediators such as TNF-α, IL-6, and IL-17, AT-MSC-MVs also exert regulatory effects on IL-12 signaling, making them particularly suitable for treating IBD subtypes characterized by immune dysregulation [43,44,45,46]. Umbilical cord-derived MSC-MVs (UmC-MSC-MVs), on the other hand, are notable for their low immunogenicity, inherent immune privilege, and superior homing capability to inflamed tissues [47]. They exhibit enhanced tissue-repair efficacy, partly mediated by miRNA cargos such as miR-378a, which inhibit NLRP3 inflammasome activation and downstream caspase-1 cleavage, thereby preventing PANoptosis and promoting cell survival [48]. These properties make UmC-MSC-MVs especially advantageous in addressing IBD phenotypes dominated by epithelial barrier disruption.

In addition to MSC-MVs, MVs originating from immune cells also play pivotal roles in intestinal disorders, primarily by modulating innate immune responses and exerting localized anti-inflammatory effects. Neutrophil-derived MVs (PMN-MVs) have been shown to inhibit the maturation of dendritic cells while promoting the secretion of TGF-β1, thereby interrupting the amplification of immune cascades and demonstrating considerable immunoregulatory potential [49]. Meanwhile, macrophage-derived MVs (MMVs) exhibit an intrinsic capacity to home to inflamed mucosal tissues and actively regulate macrophage polarization. Specifically, they attenuate proinflammatory signaling by reducing the expression of M1-associated cytokines such as IL-1β and TNF-α, while concurrently enhancing the immunosuppressive profile of M2 macrophages. Through these dual effects, MMVs contribute significantly to the resolution of local inflammation and the promotion of mucosal repair processes [50].

Moreover, milk-derived MVs (milk-MVs) have garnered increasing attention in recent years due to their non-invasive collection, inherent biosafety, and remarkable stability in the gastrointestinal environment, making them particularly well-suited for oral drug delivery applications [51]. These vesicles naturally encapsulate a range of anti-inflammatory microRNAs, including miR-148a and let-7b, which can exert therapeutic effects through multiple mechanisms—such as modulating intestinal immune cell polarization, attenuating oxidative stress, and maintaining microbial homeostasis [52]. Owing to these properties, milk-MVs represent a promising platform for the non-parenteral treatment of chronic inflammatory diseases like IBD.

In summary, MDMVs exhibit distinct functional advantages in immune regulation, resolution of inflammation, and tissue repair. MSC-MVs are particularly well-suited for systemic immunomodulation and mucosal regeneration, whereas MVs from immune cells demonstrate superior efficacy in targeting inflamed sites and rapidly correcting immune dysregulation. In contrast, milk-MVs offer a safe and non-invasive alternative, with excellent suitability for oral administration, making them an attractive option for long-term management of chronic inflammatory conditions.

**Table 2:** Mechanism of MDMVs from different sources in DSS-induced IBD.

Origin	Disease	Cargo	Target	Function	Ref.
hucMSCs	DSS-induced IBD	miR-378a-5p	NLRP3	Inhibited NLRP3 inflammasome activation, reduced ASC and caspase-1, suppressed IL-1β and IL-18	[53]
hucMSCs	DSS-induced IBD	N.D.	inflammatory cytokines, macrophages	Reduced TNF-α/IL-1β/IL-6/IL-7/iNOS, increased IL-10, decreased M1 macrophages, improved epithelial proliferation	[54]
MSCs	TNBS-induced IBD	miR-378a-3p	GATA2, AQP4, PPAR-α	Suppressed GATA2/AQP4/PPAR-α signaling, reduced apoptosis, improved tissue integrity	[55]
hucMSCs	DSS/TNBS-induced IBD	TSG-6	tight junction, T cells, pro-inflammatory cytokines	Enhanced tight junctions, inhibited Th17 and pro-inflammatory cytokines, promoted Th2/anti-inflammatory cytokines, improved barrier	[56]
hADSC	DSS-induced IBD	N.D.	ISCs, inflammatory cytokines	Promoted Lgr5^+^ ISC regeneration, reduced pro-inflammatory cytokines, increased IL-10/IL-13, protected barrier	[48]
hucMSCs	DSS-induced IBD	miR-326	NEDD8, NF-κB	Inhibited NEDD8-Cullin1 binding, reduced neddylation-related enzymes (NAe1/Uba3/UBC12F/DCNL1), suppressed NF-κB, downregulated IL-1β/IL-6	[57]
BMSCs	DSS-induced IBD	N.D.	macrophage, inflammatory cytokines	Promoted M2 polarization, reduced M1 cytokines, inhibited JAK1/STAT1, activated STAT6	[58]
HucMSCs	DSS-induced UC	miR-23b-3p	Nrf2 pathway	Activated Nrf2, increased GPX4, reduced ROS/iron/lipid peroxidation, suppressed IL--1β/IL-6	[59]
hPMSCs	TNBS-induced IBD	N.D.	inflammatory cytokines, ROS, MPO, apoptosis proteins, tight junction, MMPs	Reduced ROS/MPO/cytokines, inhibited apoptosis, upregulated ZO-1/Claudin-1/Occludin), increased IL-10/TGF-β, improved epithelium	[60]
hucMSCs	DSS-induced IBD	N.D.	METTL3-Slc37a2-YTHDF1 axis, macrophage, inflammatory cytokines	Upregulated METTL3-m6A, stabilized Slc37a2, promoted M2 macrophages, suppressed cytokines, enhanced barrier	[61]
Bovine milk	DSS-induced IBD	N.D.	gut microbiota, T-AOC	Enriched Roseburia, enhanced T-AOC, restored gut diversity	[62]
Bovine milk	DSS-induced IBD	N.D.	TLR4-NF-κB, NLRP3, Treg/Th17, microbiota, inflammatory cytokines	Suppressed TLR4-NF-κB/NLRP3, rebalanced Treg/Th17, increased *Akkermansia/Enter-orhabdus*, decreased *Desulfovibrionaceae/Enterococcaceae*, reduced IL-1β/TNF-α/IL-6/IL-17A, increased IL-10	[63]
milk	DSS-induced IBD	N.D.	TNFAIP3, COX-2, microbiota, tight junction, inflammatory cytokines	Increased *Lachnospiraceae/Rumi-nococcaceae*, suppressed NF-κB via TNFAIP3, restored ZO-1, reduced IL-6/IL-1β/TNF-α	[64]
milk	DSS-induced IBD	TGF-β1	inflammatory cytokines, DNMT1/3	Increased miR-320/miR-375/let-7a, downregulated DNMT1/3, increased TGF-β1, reduced IL-6/TNF-α	[65]
M2b macrophages	DSS-induced IBD	CCL1	CCR8	Promoted Th2/Treg, reduced IL-1β/IL-6/IL-17A	[66]

#### 2.2.2. BMVs

Unlike vesicles derived from other origins, BMVs not only regulate the intestinal immune system but also modulate the gut microbiota and influence the integrity of the intestinal barrier [67,68]. According to this, they play a multifaceted regulatory role in the interaction between the microbiota and the host, acting as crucial mediators. Based on their functional characteristics, BMVs can be broadly classified into three categories: probiotics, commensals, and opportunistic pathogens [69,70]. Each type of BMV exhibits unique biological effects in regulating host immune responses and maintaining the integrity of the intestinal barrier. Relevant examples are summarized in Table 3.

BMVs derived from probiotic strains, such as *Escherichia coli* Nissle 1917 (EcN), have been recognized as key functional carriers underpinning their antimicrobial and anti-inflammatory activities. EcN-derived outer membrane vesicles (OMVs), with diameters ranging from approximately 20 to 250 nm, can activate NF-κB and AP-1 signaling pathways in intestinal epithelial cells, thereby inducing the secretion of human β-defensin-2 (hBD-2) [71]. This process markedly enhances mucosal innate immune defenses and effectively suppresses pathogenic bacterial colonization [72,73]. Additionally, in vitro studies have shown that EcN-OMVs can trigger epithelial cell apoptosis via the PI3K/PTEN/AKT pathway, suggesting their bidirectional regulatory potential in promoting barrier repair while also contributing to epithelial renewal and homeostasis under specific conditions [74]. BMVs derived from *Lactobacillus* species also play a critical role in sustaining intestinal health. These vesicles are enriched with various proteins, DNA, and RNA, enabling them to modulate the composition of commensal microbiota, promote the production of anti-inflammatory cytokines, inhibit the proliferation of potentially pathogenic bacteria, and upregulate the expression of tight junction proteins. Collectively, these properties have been demonstrated to significantly attenuate inflammatory responses and mucosal injury in multiple IBD animal models [75].

BMVs derived from commensal anaerobic bacteria, such as *Clostridium butyricum*, have been shown to exert anti-inflammatory effects by regulating innate immune cell phenotypes. Specifically, *C. butyricum*-derived BMVs markedly upregulate the expression of M2 macrophage markers Arg1 and IL-10 in colonic tissues, thereby promoting polarization toward an anti-inflammatory phenotype and effectively alleviating DSS-induced colitis in mice [76]. Similarly, OMVs secreted by *Bacteroides fragilis* interact with dendritic cells in a TLR2-dependent manner to induce regulatory T cell (Treg) differentiation and IL-10 production, thereby enhancing mucosal immune tolerance and suppressing the progression of inflammation [77]. Notably, in genetically susceptible individuals, such as those with an ATG16L1 deficiency, this OMV-mediated immune remodeling offers a promising strategy for correcting Treg dysfunction [78].

BMVs released by certain opportunistic pathogens, including virulence-associated *E. coli* strains, exert complex effects within the intestinal microenvironment. Studies have demonstrated that *E. coli*-derived vesicles, which are enriched in respiratory chain complexes, can reduce luminal oxygen tension, re-establish anaerobic conditions, inhibit the overgrowth of Enterobacteriaceae under aerobic conditions, and mitigate DSS-induced colonic inflammation [79]. Although such effects may confer partial protective benefits, their overall impact on mucosal immunity requires further investigation to fully assess potential risks.

Overall, BMVs play a pivotal role in the onset and progression of IBD by engaging multiple targets and regulating various pathways. Compared to other sources of MVs, BMVs offer unique advantages, particularly in immune modulation and microbial–host interactions. They can effectively influence host immune responses, modulate gut microbiota, and provide significant therapeutic benefits in IBD. However, BMVs also present certain limitations, particularly due to the potential presence of endotoxins, which can trigger undesirable immune responses.

**Table 3:** Mechanism of BMVs from different sources in DSS-induced IBD.

Origin	Disease	Cargo	Target	Function	Ref.
*Roseburia intestinalis*	DSS-induced colitis	IPI	DPP4	Suppressed DPP4 activity, increased GLP-1, downregulated NFκB/STAT3 via PI3K, promoted *Bifidobacterium*	[80]
EcN 1917	DSS-induced colitis	N.D.	TLRs	Suppressed IL-1β/TNF-α/IL-6/IL-17, reduced MMP-9/COX-2/iNOS, increased TFF-3	[81]
*Clostridium butyricum*	DSS-induced UC	miR-199a-3p	Map3k4	Suppressed MAPK/NF-κB, reduced IL-6/TNF-α/IL-1β, decreased	[82]
*Lactobacillus kefiranofaciens*	TNBS-induced IBD	N.D.	NF-κB p65	Inhibited NF-κB p65, reduced IL-8, decreased MPO	[83]
*Odoribacter splanchnicus*	DSS-induced IBD	N.D.	NLRP3	Suppressed NLRP3, increased IL-10, upregulated ZO-1/Occludin, reduced apoptosis	[84]
*Lactobacillus plantarum*	DSS-induced UC	N.D.	Gut microbiota	Reduced Proteobacteria, increased *Bifidobacteria/Muribaculaceae/Akkermansia*, downregulated IL-6/IL-1β/TNF-α/IL-2, inhibited TLR4/MyD88/NF-κB	[85]
*Faecalibacterium prausnitzii*	DSS-induced colitis	N.D.	NF-κB and MAPK signaling pathways	Suppressed NF-κB/JNK/P38 (MAPK), regulated Nrf2/HO-1, upregulated ZO-1/Occludin, reduced IL-1β/IL-2/IL-6/TNF-α/IFN-γ/GM-CSF, increased IL-4/IL-10/TGF-β	[86]
*Bacteroides fragilis*	TNBS-induced colitis	PSA	TLR2	Induced TLR2, upregulated Gadd45α, promoted CD4^+^CD25^+^Foxp3^+^ Tregs, suppressed TNF-α/IL-17/IFN-γ/IL-6	[77]
*Akkermansia muciniphila*	DSS-induced colitis	N.D.	Tight junction proteins	Suppressed NO/TNF-α/L-1β/IL-6, upregulated ZO-1/MUC2, increased IgA and sIgA, reduced DAO/D-LA, increased *Firmicutes*, and decreased *Proteobacteria*	[87]
*Bacteroides fragilis*	DSS-induced colitis	miR-5119	PD-L1	Delivered miR-5119 to inhibit PD-L1, suppressed GSDMD-mediated NET, promoted Lgr5^+^ intestinal stem cells	[88]
*Clostridium butyricum*	DSS-induced UC	N.D.	Mcrophages	Promoted M2 macrophage polarization, reduced M1 macrophage infiltration, decreased *Helicobacter/Escherichia-Shigella* abundance; increased *Lactobacillus/Akkermansia/Bacteroides*, upregulated MUC2/ZO-1	[76]
*Lactiplantibacillus plantarum*	DSS-induced colitis	small RNAs (<200 nt)	Intestinal epithelial HT29 cells, pro-inflammatory cytokine IL-8	Delivered small RNAs into HT29 cells, suppressed IL-8, reduced neutrophil accumulation in colonic lamina propria	[89]
*L. rhamnosus GG*	DSS-induced UC	N.D.	TLR4-NF-κB-NLRP3 signaling pathway	Suppressed TLR4-NF-κB-NLRP3 axis, reduced TLR4/MyD88/p65/p-p65/NLRP3/ASC, decreased TNF-α/IL-1β/IL-6/IL-2), reduced *Helicobacter/Escherichia-Shigella*, increased *Lachnospiraceae/Akkermansia*	[90]
*Lactobacillus kefirgranum* PRCC-1301	DSS-induced colitis	N.D.	NF-κB signaling pathway	Suppressed NF-κB by reducing phosphorylated NF-κB p65 and phosphorylated IκBα, decreased IL-2/IL-8/TNF-α, upregulated ZO-1/claudin-1/occludin	[91]

#### 2.2.3. PDMVs

PDMVs, as naturally sourced nanovesicles, offer numerous significant advantages, making them highly promising for the treatment of IBD and related disorders [92,93,94]. PDMVs exhibit excellent biocompatibility, non-immunogenicity, high stability, and a prolonged circulation time, which enables them to effectively avoid degradation during drug delivery, ensuring sustained release of therapeutic agents [95,96,97]. Relevant examples are summarized in Table 4. Compared to synthetic membrane vesicles, their production process is simpler and more cost-effective. Furthermore, due to their natural membrane structure, PDMVs offer distinct advantages in oral drug delivery, overcoming challenges faced by traditional drug delivery systems, such as poor water solubility and low bioavailability.

PDMVs are rich in various bioactive components, including lipids, proteins, and microRNAs, which play a crucial role in modulating immune responses and inflammatory cascades. For example, ginger-derived MVs, which are abundant in lipids, proteins, and microRNAs, can effectively traverse the intestinal barrier and be internalized by intestinal epithelial cells and macrophages. This process leads to the downregulation of pro-inflammatory mediators, enhancement of antioxidant responses, and promotion of epithelial regeneration, significantly improving intestinal inflammation [98]. Similarly, turmeric-derived MVs (such as TNVs and TDNPs) exhibit potent anti-inflammatory activity in DSS-induced colitis models. Oral administration of TNVs results in selective accumulation in inflamed colonic tissues, modulation of gut microbiota composition, restoration of mucosal barrier integrity, and promotion of M2 macrophage polarization, collectively ameliorating the severity of colitis [99]. TDNPs significantly inhibit the expression of pro-inflammatory cytokines (TNF-α, IL-6, IL-1β), upregulate antioxidant genes like HO-1, and suppress NF-κB signaling pathway activation. Notably, TDNPs also demonstrate exceptional targeting capabilities, being efficiently internalized by macrophages and epithelial cells in inflamed tissues, highlighting their dual immune modulation and mucosal protection properties [100].

Additionally, tea- and blueberry-derived membrane vesicles have shown significant antioxidant advantages. Tea-derived nanovesicles, rich in polyphenols and specific microRNAs, have been shown to alleviate oxidative stress in epithelial cells, thereby inhibiting inflammatory pathways and maintaining gut homeostasis [10]. Ginseng-derived MVs (GDMVs) exert antioxidant effects by activating the p62/Nrf2/Keap1 pathway. GDMVs effectively scavenge ROS in immune cells and intestinal epithelial cells, reducing oxidative stress, and enhance the antioxidant response by promoting the nuclear translocation of Nrf2. Upon activation, Nrf2 upregulates the expression of downstream antioxidant genes, such as HO-1 and GCLC, thereby maintaining cellular redox homeostasis and protecting cells from oxidative damage. GDMVs further enhance the antioxidant capacity of cells by increasing the activity of antioxidant enzymes, effectively reducing cellular damage induced by oxidative stress. In addition, GDMVs mitigate chronic intestinal inflammation by inhibiting the TLR4/MAPK signaling pathway, significantly reducing the expression of pro-inflammatory cytokines such as TNF-α, IL-6, and IL-1β, thus exerting their anti-inflammatory effects [101].

In summary, PDMVs, due to their superior biological properties, are particularly well-suited for oral drug delivery and the treatment of chronic inflammatory diseases. Compared to BMVs, PDMVs avoid the immune reactions induced by bacterial endotoxins, offering superior safety. However, PDMVs still face limitations in drug loading capacity and targeting ability, which need to be further optimized to enhance their clinical application effectiveness.

**Table 4:** Mechanism of PDMVs from different sources in DSS-induced IBD.

Origin	Disease	Cargo	Target	Function	Ref.
Garlic	DSS-induced colitis	N.D.	TLR4/MyD88/NF-κB signaling pathway, tight junction proteins, gut microbiota, pro-inflammatory cytokines	Suppressed TLR4/MyD88/NF-κB by han-miR3630-5p targeting TLR4 3′ UTR, reduced TLR4/MyD88/NF-κB p65, upregulated ZO-1/occluding/claudin-1, increased *Lachnospiraceae*, decreased *Helicobacter/Escherichia–Shigella/Akkermansia*, reduced IL-6/IL-1β/TNF-α/IFN-γ/IL-17A/NO	[102]
Lemon	DNBS-induced colitis	N.D.	NF-κB signaling pathway, Nrf2 antioxidant pathway, inflammatory cytokines, gut microbiota	Suppressed NF-κB, reduced IL-6/TNF-α, activated Nrf2 antioxidant pathway, reduced *Pygmaiobacter/Lachnospiraceae* UCG-010*/Tuzzerella/Anaerofilum/Enteractinococcus/Acetatifactor*, increased *Enterococcus/Bacteroides_pectinophilus group/Lachnospiraceae*	[103]
*Lycium barbarum*	DSS-induced UC	Phosphatidylcholine, vitexin-2-O-rhamnoside	Inflammatory cytokines, tight junction proteins, MPO	Suppressed TNF-α/IL-12, upregulated IL-10, increased occluding/ZO-1, reduced MPO	[104]
Turmeric	DSS-induced UC	N.D.	Inflammatory cytokines, macrophages, tight junction proteins, gut microbiota	Suppressed TNF-α/IL-6/MCP-1, reduced CD16/32, increased CD206, upregulated ZO-1/occluding/E-cadherin, increased *Akkermansia/Lactobacillus, decreased Escherichia-Shigella/Helicobacter*	[99]
Turmeric	DSS-induced UC	N.D.	Inflammatory cytokines, antioxidant gene, NF-κB signaling pathway, tight junction protein	Suppressed NF-κB, reduced TNF-α/IL-6/IL-1β, upregulated HO-1, increased E-cadherin	[100]
Tea leaf	DSS-induced IBD	N.D.	Inflammatory cytokines, ROS, antioxidant enzyme, tight junction proteins, gut microbiota	Suppressed TNF-α/IL-6/IL-12, increased IL-10, reduced ROS, upregulated HO-1/GSH, decreased MDA/MPO, upregulated ZO-1/MUC2, reduced *Oscillibacter/elicobacter*, increased *Lachnospiraceae/kkermansia*	[10]
Mulberry bark	DSS-induced colitis	HSPA8	Aryl hydrocarbon receptor (AhR), COP9 Constitutive Photomorphogenic Homolog Subunit 8 (COPS8), CUL1, anti-microbial peptides, tight junction protein, gut microbiota	Activated AhR signaling pathway via HSPA8 binding, upregulated COPS8, promoted deneddylation of CUL1, induced anti-microbial peptides secretion, upregulated ZO-1, reduced *Proteobacteria/Segmented Filamentous Bacteria*, increased *Firmicutes*	[105]
Aloe	DSS-induced UC	N.D.	Oidative stress markers, tight junction proteins	Reduced p-NF-κB and p-IκB, decreased TNF-α/NO/COX2/3-NT, upregulated ZO-1/claudin4/occluding/E-cadherin/γ-catenin/α-tubulin	[106]
Grape	DSS-induced colitis	N.D.	Intestinal stem cells (Lgr5^+^), Wnt/β-catenin signaling pathway	Activated Wnt/β-catenin signaling (phosphorylated GSK-3β, nuclear translocation of β-catenin), upregulated intestinal stem cell markers (Lgr5, BMI1), promoted Lgr5^+^ stem cell proliferation	[107]
Ginseng	DSS-induced UC	Ginsenosides	Inflammatory cytokines, NF-κB signaling pathway, gut microbiota	Suppressed NF-κB signaling pathway activation, reduced TNF-α/IL-6/IL-17A, increased IL-10, decreased *Firmicutes/Bacteroidota* ratio, increased *Lactobacillus*, decreased *Helicobacter/Ruminococcus*	[108]
Broccoli	DSS-induced colitis	N.D.	Dendritic cells, AMP-activated protein kinase (AMPK), mTOR signaling pathway, inflammatory cytokines	Activated AMPK in DCs, reduced phosphorylation of p70S6K and S6, reduced TNF-α/IL-12/IFN-γ/IL-17A, increased IL-10/TGF-β	[109]
Ginseng	DSS-induced IBD	Small RNAs, ginsenosides	IKK/IκB/NF-κB signaling pathway	Activated autophagy (upregulated LC3/Atg7/Beclin1, downregulated p-mTOR/p-AKT), increased CD206/Arg1, decreased CD86/iNOS, reduced P-IKKα/β/P-IκBα/NF-κB, reduced TNF-α/IL-1β/IL-6, increased IL-10, increased ZO-1/occludin	[110]
Ginseng	DSS-induced IBD	N.D.	TLR4/MAPK signaling pathway, p62/Nrf2/Keap1 pathway	Suppressed TLR4/MAPK activation by reducing phosphorylation of ERK/JNK/p38, activated p62/Nrf2/Keap1 pathway to increase HO-1/GCLC/GCLM, decreased TNF-α/IL-6/IL-1β, enhanced ZO-1/occludin/claudin-1, promoted intestinal stem cell proliferation via Wnt/β-catenin, and reduced *Firmicutes/Bacteroidetes* ratio	[101]

#### 2.2.4. Liposomes

Liposomes have emerged as a versatile and promising nanocarrier platform for the treatment of IBD, offering unique advantages in drug protection, targeted delivery, and controlled release. Owing to their biocompatible phospholipid bilayer structures, liposomes can encapsulate both hydrophilic and hydrophobic therapeutic agents, thereby enhancing their bioavailability and stability in the harsh gastrointestinal environment. For instance, Xian et al. developed an oral liposomal formulation of an activatable budesonide prodrug, which demonstrated remarkable efficacy in experimental colitis models. The lipidation strategy conferred high stability under acidic gastric conditions while enabling site-specific drug release in inflamed intestinal tissues, leading to significantly improved remission of colitis and reduced systemic toxicity compared to free budesonide administration [28]. Beyond budesonide, a range of therapeutic agents, including corticosteroids, immunosuppressants, and biological molecules, have been successfully incorporated into liposomal carriers to optimize pharmacokinetics and minimize off-target effects.

Liposome can also be designed for both passive and active targeting of inflammatory mucosal sites, utilizing an enhanced permeability and retention (EPR) effect and ligand–receptor interactions to increase local accumulation of the drug. For instance, Rahman et al. reviewed cationic and polyethylene glycol (PEG)-modified liposomes, which further improved circulation times and facilitated the targeted delivery of anti-inflammatory drugs and immunomodulators to IBD lesion areas [26].

Overall, liposomal-based drug delivery systems have shown great potential in improving the safety, efficacy, and patient compliance of IBD therapies. Compared to naturally derived MVs, liposomes offer a simpler, more controllable production process suitable for large-scale manufacturing, while avoiding the ethical concerns and immune rejection associated with stem cell-derived vesicles. In comparison to polymersomes, liposomes have clear advantages in terms of biocompatibility and safety. While polymersomes offer greater flexibility in targeting and drug delivery control, their biocompatibility is inferior. The natural structure and biodegradability of liposomes make them a safe and effective platform for drug delivery. However, liposomes also have some limitations. Despite their advantages in biocompatibility and drug stability, their drug loading capacity and targeting ability are relatively weaker, particularly for certain types of macromolecular drugs. Furthermore, the surface modification of liposomes and selection of targeting ligands still require optimization to enhance their therapeutic efficacy for specific lesions.

#### 2.2.5. Polymersomes

Polymersomes have emerged as a compelling platform for drug delivery in IBD, owing to their highly tunable physicochemical properties, excellent biocompatibility, and superior stability in the complex gastrointestinal environment. Polymersomes, typically self-assembled from amphiphilic block copolymers, possess greater mechanical strength and membrane integrity compared to conventional liposomes. This structural advantage allows them to encapsulate both hydrophilic and hydrophobic therapeutic agents while enabling multi-functional targeting and controlled release strategies [31].

Tollemeto et al. developed a pH-responsive polymersome by incorporating carboxyl functional groups into the block copolymer, which ensured structural stability under gastric acidic conditions while achieving site-specific release of immunosuppressive agents, such as mycophenolate mofetil, in the near-neutral pH environment of the small intestine [29]. In vitro and in vivo studies demonstrated that this polymersome maintained its integrity during gastric transit, thereby preventing premature drug leakage, and rapidly disassembled in the intestinal lumen, significantly enhancing drug accumulation at the target site. This approach holds promise for safer and more effective immunosuppressive therapy in patients with IBD.

In addition, polymersomes have been applied as nanocarriers to encapsulate natural antioxidants and bioactive compounds for multi-target modulation of the intestinal inflammatory microenvironment. Zhong et al. summarized advances in polymer-based nano-delivery systems for the encapsulation of anthocyanin and reported that such systems not only markedly improve the solubility and bioavailability of anthocyanins but also exert robust anti-inflammatory effects in multiple IBD models through targeted regulation of NF-κB signaling pathways, modulation of the gut microbiota, and suppression of oxidative stress [30].

In conclusion, polymersomes hold tremendous potential for the treatment of IBD owing to their customizable physicochemical properties and multifunctional stimulus-responsive release capabilities. Compared with naturally derived MVs, polymersomes offer the advantage of precisely controllable synthesis, thereby avoiding the ethical concerns and potential immune rejection issues associated with stem cell-derived vesicles. Relative to liposomes, the most prominent strengths of polymersomes lie in their superior stability and enhanced structural integrity [111,112,113]. While liposomes are widely used for the delivery of hydrophilic drugs and siRNA, they often suffer from suboptimal stability, particularly in vivo, where premature drug leakage may occur [114]. As structural analogues of liposomes, polymersomes exhibit greater mechanical robustness, enabling prolonged stability in the gastrointestinal environment and mitigating the risk of premature payload release. Nevertheless, a notable drawback of polymersomes compared to liposomes is their lower biodegradability, which may raise concerns regarding long-term safety in vivo. Therefore, while polymersomes demonstrate clear advantages in stability, drug loading capacity, and controlled release, further optimization of their biodegradability is warranted to maximize their clinical therapeutic potential.

#### 2.2.6. EBMVs

EBMVs, as a new generation of nanoscale carrier platforms, strategically integrate the tunable physicochemical properties of synthetic nanomaterials with the targeting specificity and immunomodulatory capacity of natural cell membranes. Unlike conventional liposomes or polymersomes, EBMVs incorporate key membrane proteins—such as adhesion molecules, integrins, and immunoregulatory receptors—derived from source cells into the synthetic vesicle scaffold, thereby endowing them with highly selective bio-recognition and targeting capabilities [115]. This “hybrid” design enables both active homing to inflamed intestinal tissues and modulation of local immune responses, while retaining the structural stability, scalable manufacturing potential, and compositional controllability of synthetic carriers. A representative example is provided by the work of Corbo et al. [33], who stimulated murine leukocytes with retinoic acid to upregulate the expression of α4β7 integrin, extracted the membrane proteins, and co-assembled them with phospholipids and cholesterol to produce specialized “leukosomes.” In a DSS-induced colitis model, these reconstituted vesicles selectively accumulated at sites of inflammation, significantly suppressed the expression of pro-inflammatory cytokines (TNF-α and IL-6), and promoted mucosal healing [116,117,118]. Notably, these therapeutic effects were achieved without loading any additional pharmacological payloads, highlighting the intrinsic bioactivity conferred by the membrane proteins themselves.

EBMVs combine the controllable physicochemical characteristics, reproducible manufacturing, and high structural robustness of synthetic nanocarriers with the homologous targeting, immune evasion, and inherent bioactivity of natural cell membranes. This dual functionality positions them as a highly customizable and rationally designable platform for precision therapy in IBD, particularly in scenarios requiring both targeted delivery and inflammation microenvironment-specific immunomodulation [119]. Moreover, their modular assembly strategy allows for flexible selection of membrane sources and fine-tuning of membrane protein composition to match disease-specific therapeutic requirements.

Compared with naturally derived MVs, EBMVs circumvent batch-to-batch variability, poor scalability, and the ethical or immunogenicity concerns associated with certain cell sources, while preserving the bio-recognition and targeting features of native vesicles. Nevertheless, challenges remain in the efficient extraction of membrane proteins, maintenance of their functional integrity, and their stable integration into synthetic scaffolds. Overcoming these technical bottlenecks—through advances in membrane separation techniques, protein stabilization methods, and nano-assembly engineering—will be essential to fully realize the translational potential of EBMVs in clinical IBD therapy.

## 3. Therapeutic Mechanism of MV-DDSs in IBD

**Figure 2:**
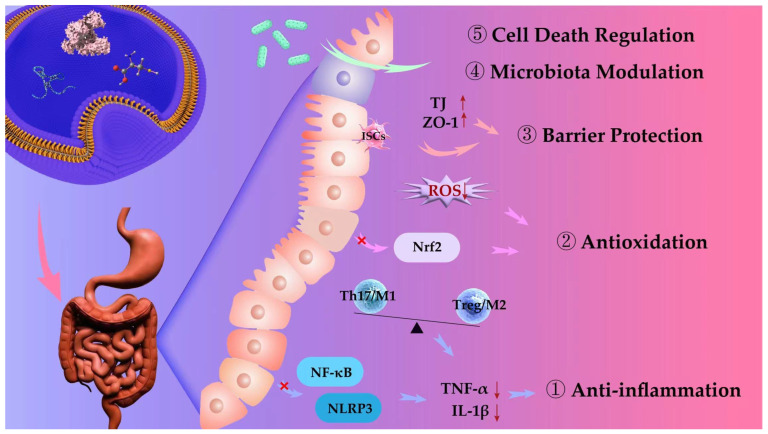
Therapeutic effect of MV-DDSs in IBD. In this figure, red arrows are used to denote the change trends of target substances: upward red arrows stand for an increase, and downward red arrows signify a decrease.

MV-DDSs demonstrate significant therapeutic potential for IBD, attributable to their unique biological properties and payload. Their therapeutic efficacy in IBD is manifested through five principal mechanisms: anti-inflammatory and antioxidant effects, protection of the intestinal mucosal barrier, modulation of gut microbiota, and regulation of programmed cell death pathways (Figure 2).

### 3.1. Anti-Inflammation

**Figure 3:**
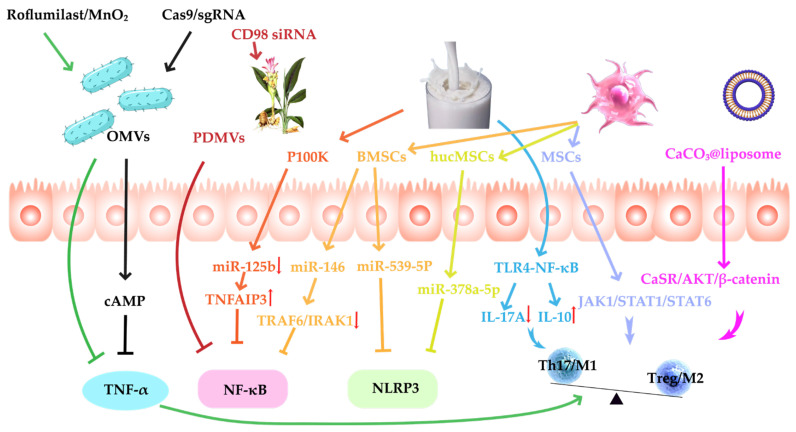
Anti-inflammatory mechanism of MV-DDSs in IBD. Colors of lines are used to distinguish pathways of MV-DDSs from different sources.

IBD is a chronic, recurrent disease driven by immune dysregulation of the intestinal mucosa. Pro-inflammatory cytokines such as TNF-α and IL-1β are significantly elevated in IBD patients. These cytokines not only directly mediate damage to intestinal epithelial cells and disruption of intestinal barrier function but also activate key inflammatory networks such as the NF-κB signaling pathway and the NLRP3 inflammasome, triggering inflammatory cascades and forming a vicious positive feedback loop that further amplifies the intensity of the immune response and pathological damage. In addition to local inflammatory cytokine storms, the chronic progression of IBD is also significantly influenced by immune imbalance. This manifests as dysregulation between innate and adaptive immune systems, such as weakened Treg function, excessive expansion of Th17 cells, or sustained maintenance of the M1 pro-inflammatory phenotype by macrophages. This imbalance not only delays the resolution of inflammation but also impedes tissue repair and the reconstruction of immune tolerance. Therefore, inhibiting the abnormal expression of key inflammatory factors, blocking their upstream signaling pathways, and restoring the homeostasis and tolerance mechanisms of the immune system are considered the three core strategies for alleviating IBD activity, slowing disease progression, and achieving long-term clinical remission (Figure 3).

#### 3.1.1. Inhibition of Pro-Inflammatory Cytokines

In IBD, pro-inflammatory cytokines such as TNF-α, IL-1β, and IL-6 activate immune cells, promote inflammatory responses, and disrupt the intestinal epithelial barrier, leading to the loss of intestinal function and pathological changes [120]. MVs, by carrying specific cargo molecules, can effectively modulate the intestinal inflammatory response and reduce the release of pro-inflammatory cytokines, thus playing a significant role in the treatment of IBD.

TNF-α, as a key pro-inflammatory factor in IBD, not only promotes the activation and migration of immune cells but also induces damage to intestinal epithelial cells, disrupting the intestinal barrier and exacerbating the inflammatory response. According to the relevant literature, OMVs derived from EcN 1917 can accumulate in the intestinal mucosa through pathogen-associated molecular patterns (PAMPs) and, to some extent, inhibit the overexpression of TNF-α [121]. Although this intrinsic anti-inflammatory effect is limited, it still holds potential for alleviating IBD. To enhance the anti-inflammatory effects of OMVs, Song [122] et al. loaded phosphodiesterase 4 (PDE4) inhibitor roflumilast and MnO_2_ nanoparticles into OMVs. This combination not only upregulated cAMP synthesis and enhanced the anti-inflammatory response but also significantly suppressed the production of TNF-α and improved intestinal mucosal repair. This strategy, by altering the cargo and delivery mechanism of OMVs, significantly enhanced their therapeutic efficacy in regulating TNF-α. Furthermore, Lin [6] et al. coupled Cas9/sgRNA ribonucleoprotein nanoclusters to OMVs, enabling direct transcriptional disruption of the TNF-α gene. This approach significantly downregulated the expression of TNF-α, IL-1β, and IL-6, while upregulating IL-10. Through this gene-editing method, the anti-inflammatory effects of OMVs were further strengthened, allowing for precise targeting of pro-inflammatory factors and enhancing therapeutic outcomes. This strategy provides a more durable and stable solution for IBD treatment. Therefore, MVs-DDSs precisely regulate the release of pro-inflammatory cytokines by carrying either naturally occurring or exogenously loaded molecular cargo, thereby effectively alleviating the inflammatory response in IBD.

#### 3.1.2. Suppression of Inflammatory Signaling Pathways

In IBD, key signaling pathways such as NF-κB and NLRP3 inflammasome are widely recognized as central molecular hubs driving the amplification of persistent inflammation and hindering the intestinal epithelial barrier’s repair [123,124]. These pathways play a decisive role in mediating the release of pro-inflammatory factors, cytokine cascade amplification, and immune dysregulation, making them critical targets in current inflammation intervention research.

NF-κB is a pivotal transcription factor that orchestrates the expression of pro-inflammatory mediators, including TNF-α, IL-1β, and IL-6. Its sustained activation creates a self-reinforcing inflammatory loop that significantly exacerbates intestinal inflammation. In the context of IBD, aberrant hyperactivation of the NF-κB pathway is a central mechanism underlying the epithelial barrier’s disruption, immune dysregulation, and chronic inflammation. Consequently, precise modulation of NF-κB activity has become a key strategy in anti-inflammatory therapeutic interventions. CD98, a transmembrane protein involved in amino acid transport and adhesion signaling, plays a critical role in facilitating upstream NF-κB activation [125]. PDMVs from ginger are enriched with natural anti-inflammatory miRNAs and bioactive small molecules. After oral administration, they effectively accumulate in the colon, suppressing CD98 expression, inhibiting NF-κB activation, and reducing the transcription and release of pro-inflammatory factors. Building on this, targeted delivery of anti-CD98 siRNA via these vesicles further silences CD98 expression, resulting in more robust NF-κB inhibition and substantial alleviation of colitis symptoms [126]. Similarly, miR-125b has been identified as a key pro-inflammatory miRNA. It enhances NF-κB signaling by repressing the translation of the anti-inflammatory protein TNFAIP3 (A20), a negative regulator of NF-κB. Milk-derived P100K MVs have been reported to effectively downregulate miR-125b, thereby relieving its inhibition on A20. This leads to a remarkable 375% increase in A20 protein expression—significantly higher than the 135% observed with P35K vesicles—ultimately resulting in suppression of NF-κB activity and attenuation of the inflammatory response [64]. In contrast to the pro-inflammatory mechanisms described above, miR-146 is widely recognized as a potent anti-inflammatory miRNA. MVs derived from BMSCs, enriched with miR-146, have demonstrated the ability to inhibit downstream NF-κB signaling by downregulating TRAF6 and IRAK1 expression and blocking phosphorylation of the NF-κB p65 subunit. This leads to a marked reduction in the production of pro-inflammatory cytokines such as TNF-α and IL-1β [36].

In addition to NF-κB, the NLRP3 inflammasome is another pivotal regulator of inflammatory responses. By sensing endogenous and exogenous signals, it activates caspase-1, which in turn cleaves and releases pro-inflammatory cytokines such as IL-1β, further exacerbating intestinal inflammation. Studies have shown that MVs derived from BMSCs primarily regulate the function of the NLRP3 inflammasome through the delivery of miR-539-5p. These BMSC-derived MVs inhibit NLRP3 activation by miR-539-5p, preventing its interaction with ASC and caspase-1, thereby reducing the release of pro-inflammatory cytokines such as IL-18 and IL-1β [127]. Similarly, hucMSC-derived exosomes deliver miR-378a-5p, which also inhibits NLRP3 activation, reduces the release of IL-1β, and alleviates colonic inflammation [53]. Collectively, these findings underscore the potent anti-inflammatory potential of MV-DDSs, which act through the precise modulation of key signaling pathways such as NF-κB and NLRP3, offering a promising therapeutic strategy for the treatment of IBD.

#### 3.1.3. Modulation of Immune Homeostasis and Mucosal Tolerance

The imbalance of the Th17/Treg cell ratio and the polarization of macrophages directly affect the intensity and duration of immune responses, thus determining the onset and control of inflammation. In IBD, excessive Th17 cell responses and the activation of M1 macrophages are key factors in sustaining intestinal inflammation. In contrast, Treg cells and M2 macrophages help suppress excessive immune responses and restore immune homeostasis. Therefore, regulating the Th17/Treg balance and macrophage polarization has become a crucial therapeutic target in IBD treatment.

Studies have shown that milk-derived MVs inhibit the TLR4-NF-κB signaling pathway and activation of the NLRP3 inflammasome, reducing the release of pro-inflammatory factors like IL-17A and suppressing Th17 cell differentiation. At the same time, they upregulate the anti-inflammatory factor IL-10 and promote the function of IL-10^+^Foxp3^+^ Treg cells, helping restore the Th17/Treg balance [63].

In addition to regulating the Th17/Treg balance, macrophage polarization is also a critical aspect of IBD treatment. Naturally-derived MVs promote macrophage polarization to the M2b phenotype, downregulating colonic inflammation, which helps alleviate experimental colitis in mice. For example, MSC-MVs promote macrophage polarization to the M2 by downregulating the JAK1/STAT1/STAT6 signaling pathway in colonic tissue [128]. Similarly, *Scutellaria baicalensis*-derived MVs, carrying miR-4371c, also promote macrophage polarization to the M2 phenotype [129]. To further enhance immune modulation, researchers have shown that TNF-α-pretreated MVs upregulate miR-24-3p expression, targeting and inhibiting IRF1, which further promotes macrophage polarization from the pro-inflammatory M1 phenotype to the anti-inflammatory M2 phenotype, thereby more effectively alleviating intestinal inflammation [130]. Furthermore, the dual-drug OMVs developed by Song [122] et al. (containing roflumilast and MnO_2_) not only promote macrophage polarization from the pro-inflammatory M1 phenotype to the reparative M2 phenotype but also amplify cAMP-mediated anti-inflammatory effects, achieving combined inflammation control and mucosal repair. In contrast to these naturally derived MVs, CaCO_3_ mineral-coated liposomes release Ca^2+^ in response to pH changes, activating the CaSR/AKT/β-catenin signaling pathway and promoting macrophage polarization to the M2 phenotype [131]. The precision control and customizability of this liposomal system hold significant potential for IBD treatment. These studies indicate that MV-DDSs regulate immune responses through various mechanisms, particularly macrophage polarization and Th17/Treg balance, providing a multidimensional and synergistic approach for IBD therapy.

### 3.2. Antioxidation

**Figure 4:**
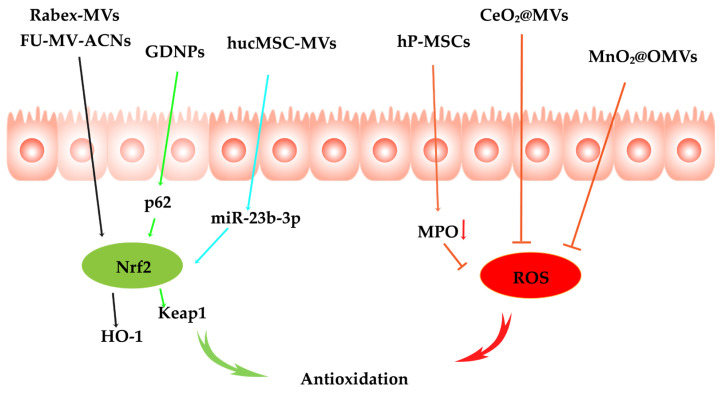
Antioxidant mechanism of MV-DDSs in IBD.

In the chronic progression of IBD, oxidative stress is widely recognized as a central mechanism driving disruption of the epithelial barrier, exacerbating mucosal inflammation, and impeding tissue repair processes. Excessive production of ROS not only directly induces apoptosis and DNA damage but also persistently activates inflammatory pathways such as NF-κB, creating a vicious cycle that underlies recurrent inflammation and tissue fibrosis. Consequently, targeted intervention in oxidative stress and effective scavenging of ROS are regarded as pivotal strategies to halt disease progression and restore intestinal homeostasis (Figure 4).

#### 3.2.1. Oxidative Stress Signaling Pathway Inhibition

Persistent oxidative stress can markedly exacerbate intestinal immune responses, compromise the integrity of the mucosal barrier, and ultimately lead to structural and functional damage to the intestinal tissue. Nuclear factor erythroid 2-related factor 2 (Nrf2) serves as a master transcription factor in the regulation of oxidative stress responses and plays a pivotal role in maintaining redox homeostasis. As a key regulator of the antioxidant defense system, Nrf2 orchestrates the expression of downstream antioxidant enzymes—such as HO-1, NQO1, and SOD—which effectively mitigate oxidative damage. Consequently, targeting the Nrf2 signaling pathway holds considerable promise for alleviating the symptoms of IBD, attenuating inflammation, and restoring immune homeostasis.

A great deal of evidence suggests that MVs play a pivotal role in attenuating oxidative stress pathways in IBD. For instance, MVs isolated from Rabex exert antioxidant effects by activating the Nrf2/HO-1 signaling pathway, significantly alleviating oxidative stress in the intestinal environment [132]. Similarly, ginseng-derived nanoparticles (GDNPs) have been shown to enhance antioxidant defenses via the p62/Nrf2/Keap1 axis. Liu [101] et al. further demonstrated that hucMSC-MVs deliver miR-23b-3p into macrophages, thereby activating the Nrf2 antioxidant pathway. Collectively, these studies highlight the therapeutic potential of MV-based delivery systems in modulating Nrf2-mediated antioxidant responses through diverse molecular mechanisms in IBD treatment. To further enhance the efficacy of bioactive compounds in oxidative-related disorders, researchers have developed functionalized MV systems to improve their stability and antioxidative performance. In a recent study, anthocyanins (ACNs) were encapsulated into milk-derived MVs using ultrasound-assisted methods. The vesicle surface was subsequently modified with fucoidan (FU) to construct a novel FU-MV-ACNs nano-delivery platform. This system not only significantly improved the gastrointestinal stability and oral bioavailability of ACNs through membrane encapsulation, but also enhanced their targeted accumulation and cellular uptake in diseased tissues due to the mucoadhesive and sustained-release properties of FU. More importantly, FU-MV-ACNs exerted strong antioxidative effects by activating the Nrf2/HO-1 pathway, which attenuated oxidative stress and effectively protected tissue cells from ROS-induced damage [133].

#### 3.2.2. ROS Scavenging

In the chronic progression of IBD, ROS are considered a driving factor of the inflammatory response. The excessive production of ROS not only directly induces cell death and DNA damage but also continuously activates inflammatory pathways, such as NF-κB, thereby creating a vicious cycle of recurrent inflammation and tissue fibrosis [134]. Therefore, effectively clearing ROS is considered a key strategy for halting disease progression and restoring intestinal homeostasis.

Recent evidence suggests that MV-DDSs play an important role in clearing ROS and have significant potential in treating IBD. Duan [60] et al. found that MVs derived from human placenta mesenchymal stem cells (hP-MSCs) effectively reduced ROS levels in mouse colon tissues by decreasing myeloperoxidase (MPO) activity. However, the ROS clearance ability of most membrane vesicles remains limited, failing to fully meet the therapeutic needs of IBD. Therefore, functionalized MV-DDSs based on nanoenzymes have gained increasing attention in recent years. Nanoenzymes, which exhibit peroxidase-like activity, can effectively clear ROS. Although turmeric-derived MVs naturally have mucosal enrichment and moderate anti-inflammatory effects, their efficacy in controlling oxidative stress has shown limitations. To overcome this, researchers have incorporated cerium oxide (CeO_2_) nanocrystals into MVs via in situ growth methods. CeO_2_ efficiently clears ROS by mimicking superoxide dismutase activity [135]. Similarly, Yue [136] et al. also incorporated CeO_2_ nanocrystals into Treg-derived MVs to further enhance their antioxidant effects. Furthermore, Li [137] et al. developed an MnO_2_@OMVs antioxidant drug delivery platform using EcN 1917 OMVs as a carrier. This system targets macrophages and intestinal epithelial cells through the PAMPs on OMVs, allowing the nanoenzyme to accumulate in the intestinal inflammation region. It effectively catalyzes the breakdown of excess H_2_O_2_ at the site of the lesion, significantly clearing ROS. In conclusion, through functionalized loading strategies, MV-DDSs can significantly enhance the ability to clear ROS and effectively modulate oxidative stress.

### 3.3. Barrier Protection

During the chronic progression of IBD, dysfunction of the intestinal barrier not only facilitates the translocation of luminal microbiota and toxins into the lamina propria—serving as a key driver of sustained mucosal immune activation—but also directly impedes epithelial regeneration and repair, thereby contributing to the relapse of patients. Therefore, promoting the expression of tight junction protein, enhancing the stability of the barrier structure, and promoting the regeneration and repair of epithelial cells are of great significance for restoring the integrity of the intestinal barrier and preventing the recurrence of diseases.

#### 3.3.1. Tight Junction Protein Expression Enhancement

The role and importance of tight junction proteins in the treatment of IBD cannot be overstated. Tight junction proteins regulate the connections between intestinal epithelial cells, maintaining the integrity of the intestinal barrier. In IBD patients, the intestinal barrier function is often compromised, leading to increased intestinal permeability and persistent chronic inflammation. Therefore, regulating the expression of tight junction proteins is critical for restoring intestinal barrier function and alleviating IBD symptoms.

Studies have shown that BMVs have unique advantages in regulating the expression of tight junction proteins. For instance, MVs derived from EcN-1917 and ECOR63 strains can significantly upregulate the expression of ZO-1 and claudin-14 while downregulating claudin-2. This results in improved barrier function and reduced permeability. Although BMC-MVs also have a baseline ability to repair the barrier, their effect remains limited. To enhance their therapeutic potential, researchers have genetically modified MSCs via lentivirus transfection to overexpress EphB2, producing EphB2-enriched MSC-MVs. This loading strategy not only upregulated the expression of ZO-1 and occludin but also significantly reduced tight junction damage in a colitis model by inhibiting the RhoA/ROCK signaling pathway [138]. In parallel, another study demonstrated that MSC-MVs enriched with miR-181a further enhanced their ability to promote the expression of epithelial tight junction proteins. Compared to unloaded vesicles, these MVs significantly elevated the levels of ZO-1, claudin-1, and occludin, highlighting the crucial role of miRNA loading in maintaining tight junction integrity [37].

#### 3.3.2. Epithelial Regeneration Promotion

Intestinal stem cells not only maintain the homeostasis of the intestinal epithelium but also accelerate the repair of intestinal damage by promoting epithelial cell regeneration when the intestinal barrier is compromised, thereby restoring intestinal function. During the chronic inflammation process of IBD, the normal proliferation and differentiation of intestinal stem cells are suppressed, which hinders the repair of the intestinal barrier. Therefore, promoting the proliferation and functional recovery of intestinal stem cells has become an important strategy for the treatment of IBD.

In this context, MV-DDSs have demonstrated significant effects. MVs derived from human amniotic fluid stem cells (hAFSCs) activate the Wnt/β-catenin signaling pathway and significantly upregulate the expression of the intestinal stem cell-specific marker Lgr5+ (compared to the NEC + PBS group, *p* < 0.0001, and the expression level was higher than the control group, *p* = 0.0003) [139]. Although MSC-MVs can promote the proliferation of Lgr5+ intestinal stem cells and epithelial cells, their efficacy in making repairs remains limited in highly inflammatory environments [48]. The cytokines and immune responses in the inflammatory environment can affect the function of HucMSC-MVs, restricting their repair effects in the local inflammatory areas. To overcome this limitation, researchers have designed a strategy in which MSC-MVs are functionalized with IL-33/IL1RL1 regulatory molecules. This effectively inhibits the activation of mast cells, reduces local inflammation, and creates a microenvironment conducive to epithelial repair [140]. This strategy enhances the application potential of MV-DDSs in intestinal repair.

### 3.4. Microbiota Modulation

During the persistent progression of IBD, intestinal dysbiosis is increasingly recognized not merely as a concomitant phenomenon but as a central driver of mucosal barrier disruption, immune dysregulation, and recurrent inflammation [141]. Historically, therapeutic strategies have primarily focused on suppressing inflammatory responses, while effective restoration of microbial homeostasis has often been overlooked—a gap that contributes to sustained expansion of pathogenic bacteria and depletion of beneficial species, ultimately compromising the durability of long-term treatment. Therefore, modulating the gut microbiota has become a key strategy in the treatment of IBD.

In recent years, MV-DDS has emerged as a promising tool for microbiota modulation due to its targeted delivery, mucosal accumulation, and the ability to encapsulate various bioactive cargos. Gu [37] et al. discovered that RNA-181a in MSC-MVs increased the abundance of lactobacilli while reducing the abundance of Bacteroides, thereby improving the gut microbiota’s composition and alleviating experimental colitis symptoms. Unlike MSC-MVs, ginger-derived MVs carry miR-167a. To enhance its effect, the researchers specifically extracted miR-167a and re-encapsulated it into ginger-derived MVs. After oral administration, these vesicles were efficiently internalized by lactobacilli and mediated the upregulation of SpaC mRNA expression, thus supporting stable and persistent colonization along the colonic mucosa. In the DSS-induced colitis model, this approach resulted in more than a twofold increase in lactobacilli abundance, a significant rise in IL-22 levels, and a nearly 40% reduction in the inflammation score, while scrambled RNA-loaded vesicles provided only limited protection under the same conditions [142]. Another study further emphasized the value of pretreatment methods. LPS-pretreated MSC-MVs selectively enhanced the proliferation of beneficial *Clostridia*_UCG 014 while reducing the colonization density of Streptococcus and Enterococcus [143]. This “preprogramming” strategy proved to be superior to unmodified vesicles in promoting microbiota homeostasis and alleviating colitis pathology. Notably, the different vesicle sources and cargo configurations appeared to play complementary roles: PDMVs primarily regulate bacterial adhesion genes and colonization, while MSC-MVs are involved in host immune signaling and reshape the nutritional microenvironment to achieve multidimensional microbiota modulation.

These studies demonstrate the potential effects of MV-DDS on regulating the intestinal microbiota. In the future, MV-DDS may enable precise regulation of the intestinal microbiota through functional design, and may also enhance the therapeutic effects of IBD by synergizing with other treatments.

### 3.5. Cell Death Regulation

Programmed cell death (PCD) pathways, including apoptosis, pyroptosis, necroptosis, PANoptosis, ferroptosis, and autophagy, play crucial roles in intracellular and extracellular signal transduction, immune response, repair mechanisms, and intestinal barrier function. In IBD, excessive or dysregulated PCD is one of the primary causes of intestinal damage, inflammation, and barrier dysfunction [144,145]. Therefore, modulating these PCD offers new strategies and directions for the treatment of IBD.

Apoptosis, as a classical form of PCD, is typically driven by the mitochondrial pathway and the activation of caspase family proteins, leading to orderly cell death. Excessive apoptosis of intestinal epithelial cells can result in the disruption of intestinal barrier function, thereby exacerbating the progression of IBD. Based on this, Li [55] et al. investigated natural MVs derived from MSCs and engineered them to enrich miR-378a-3p, constructing a delivery platform to intervene in the apoptosis of colonic epithelial cells in UC. MSC-MVs themselves exhibit certain biocompatibility and mucosal delivery capabilities, but their inhibitory effect on epithelial cell apoptosis is relatively limited. By transfecting MSCs with miR-378a-3p, they produced MVs enriched with this miRNA, significantly enhancing their ability to regulate mucosal homeostasis. Mechanistic studies revealed that miR-378a-3p-loaded MVs primarily exert their effects by downregulating the expression of GATA2, further inhibiting the upregulation of AQP4 and PPAR-α, thereby reducing intestinal epithelial cell apoptosis.

Pyroptosis is an inflammatory form of PCD activated by inflammasomes. The activation of the NLRP3 inflammasome promotes the activation of caspase-1, which subsequently cleaves Gasdermin D, leading to the rupturing of cell membrane and the release of inflammatory cytokines. miR-539-5p-enriched BMSCs-MVs block this process by targeting and inhibiting NLRP3-inflammasome. It significantly reduces IL-1β and IL-18 levels and effectively improves inflammatory lesions in DSS-induced IBD models [127]. Li [146] et al. further explored the potential of MVs derived from induced pluripotent stem cell-derived mesenchymal stem cells (iPSC-MSCs) in regulating epithelial cell pyroptosis. Although MVs were effectively internalized by intestinal epithelial cells and showed mild inhibition of pyroptosis-related proteins (such as GSDMD-N and caspase-1), their impact on pyroptosis inhibition remained limited. To enhance this effect, the researchers genetically engineered iPSC-MSCs to overexpress circ-CCND1, producing extracellular vesicles enriched with this circular RNA (MVs-oe-CCND1). Upon delivery, circ-CCND1 significantly downregulated KDM6B protein and ELF3 mRNA levels, increased H3K27me3 modification, de-repressed miR-342-3p, and established a negative feedback loop, further inhibiting KDM6B expression. Through this mechanism, MVs-oe-CCND1, compared to control vesicles, significantly reduced the levels of GSDMD-N and IL-1β, decreased pyroptosis by approximately 60%, and markedly improved the histological scoring of the intestinal mucosa in a DSS-induced colitis model.

As research advances, increasing evidence suggests that different forms of cell death are not isolated but instead interact significantly with each other. Based on these extensive signaling crosstalks, the concept of “PANoptosis” was introduced in 2019: a cell death mode driven by the PANoptosome multiprotein complex that integrates key signaling features of apoptosis, pyroptosis, and necroptosis within the same intestinal epithelial cell, providing an explanation for the comprehensive pathological features of UC mucosal damage [147,148]. As a result, PANoptosis has become a critical target in the treatment of IBD. Gong [149] et al. engineered Treg-derived MVs by loading them with selenium (Se) and modifying them with mitochondrial-targeting peptide SS-31. This system not only utilizes SS-31 for inflammation-specific release but also inhibits PANoptosis by reducing excessive ROS in macrophages, significantly alleviating inflammation and intestinal damage in a DSS-induced UC model.

Additionally, ferroptosis, an emerging form of PCD, has garnered increasing attention in recent years in the context of IBD research. Ferroptosis primarily occurs due to the excessive accumulation of intracellular iron, which catalyzes the production of ROS, leading to lipid peroxidation and subsequent cell damage. MVs derived from endometrial regenerative cells have been shown to downregulate ACSL4, a key regulator of ferroptosis, while upregulating GPX4 antioxidant activity. This effectively inhibits lipid peroxidation and ferroptosis in intestinal epithelial cells, thereby promoting mucosal repair and reducing inflammation [150].

Above the studies have revealed the complex role of PCD in IBD and its potential therapeutic value. MV-DDSs, derived from various sources, can effectively target and regulate PCD by carrying key molecules. In the future, by utilizing functionalized MV-DDSs to achieve more precise modulation of these PCDs and to enable the coordinated regulation of multiple PCDs, novel therapeutic strategies for the treatment of IBD may be developed.

## 4. Delivery Strategies of MV-DDSs in IBD

In IBD, MV-DDSs offer a diverse range of administration routes, each demonstrating distinct advantages and facing specific challenges depending on their characteristics and clinical application scenarios (Table 5). Common delivery strategies include intravenous injection, rectal administration, oral delivery, and intraperitoneal injection. Among these, oral delivery has been the most extensively investigated, particularly in the context of plant-derived vesicles and bacterial extracellular vesicles. Owing to its convenience and high patient compliance, oral administration is regarded as the preferred approach for long-term IBD management [95,151]. However, despite the ability of plant- and bacteria-derived vesicles to accumulate efficiently in the colon and suppress pro-inflammatory cytokine expression, they still face substantial degradation in the gastrointestinal environment due to gastric acid and digestive enzymes, ultimately compromising their therapeutic bioactivity [27]. To address these limitations, Deng [14] et al. developed an effective self-assembled layer-by-layer (LbL) MV delivery system for oral administration, enabling targeted release of MVs at inflamed colonic sites to improve UC therapy. In this strategy, the outer shell of MSC-MVs was engineered using biocompatible and biodegradable polymers—N-(2-hydroxy)propyl-3-trimethylammonium chitosan chloride (HTCC) and oxidized konjac glucomannan (OKGM). These materials were selected to facilitate colonic targeting while simultaneously protecting the MVs from enzymatic degradation in the upper gastrointestinal tract. Compared to intravenous delivery, the orally administered LbL-EVs achieved significantly greater therapeutic efficacy in alleviating UC symptoms, despite utilizing only half the dose of EVs.

Rectal administration delivers therapeutics directly to the colon and rectum, thereby bypassing gastrointestinal degradation and first-pass hepatic metabolism. This approach enhances local drug concentrations and improves therapeutic efficacy, making it particularly suitable for patients requiring localized treatment while minimizing systemic side effects. However, rectal delivery also presents certain limitations, primarily including the short retention time of the administered drug—due to potential expulsion by defecation or flatulence—and the incomplete coverage of the proximal colon. To address these challenges, Nie [152] et al. developed a bioadhesive microcarrier system. They encapsulated IL-27-loaded MSC-MVs within a dopamine methacrylamide (DMA)-modified hydrogel matrix, fabricated using microfluidic techniques. This hydrogel exhibited excellent mucosal adhesion properties. Following rectal administration, the catechol groups of DMA formed hydrogen bonds with amino groups on the colonic mucosal surface, ensuring firm anchoring of the formulation at the site of colonic inflammation and reducing the likelihood of drug loss through intestinal transit. Moreover, the crosslinked network structure of the hydrogel effectively protected the embedded MVs from enzymatic degradation, allowing for sustained and localized release of IL-27. This strategy significantly alleviated inflammatory responses and promoted epithelial barrier repair.

Intravenous injection, as a systemic administration route, offers the advantages of rapid onset and broad pharmacological action, making it particularly suitable for acute inflammatory episodes and systemic immunomodulation in IBD treatment. However, this route generally lacks tissue specificity, limiting drug accumulation in inflamed colonic regions and increasing the risk of off-target effects in non-diseased tissues. To overcome these limitations, Gong [149] et al. engineered regulatory Treg-MVs by decorating their surface with the mitochondria-targeting tetrapeptide SS-31. This peptide possesses a positive charge and an aromatic structure, enabling selective binding to cardiolipin located on the inner mitochondrial membrane. Through this design, the targeted delivery of MVs to mitochondria was significantly enhanced. Upon delivery, the SS-31-modified Treg-MVs effectively suppressed PANoptosis by attenuating excessive ROS.

Intraperitoneal (i.p.) injection is a commonly employed drug delivery route in experimental models of IBD. By allowing direct entry into the systemic circulation, i.p. administration avoids the destructive effects of gastric acid and digestive enzymes on vesicular structures, thereby preserving the biological activity of nucleic acid or protein cargos encapsulated within membrane vesicles. Nevertheless, as a non-targeted, systemic delivery strategy, intraperitoneal injection has inherent limitations in achieving a site-specific accumulation of drugs. To overcome this challenge and achieve precise delivery to inflamed tissues, Du [153] et al. innovatively employed M1-MVs as inflammation-homing carriers. These M1-MVs exhibit a high surface expression of the cc chemokine receptor 2 (CCR2), which specifically recognizes and binds to monocyte chemoattractant protein-1 (MCP-1), a chemokine markedly upregulated at inflammatory sites. Through the CCR2/MCP-1 axis, M1-MVs are actively recruited to regions of high MCP-1 expression, enabling inflammation-targeted accumulation. Following i.p. administration, the siIRF5 cargo delivered by M1-MVs successfully downregulated the expression of pro-inflammatory transcription factor IRF5 within macrophages, thereby inhibiting M1 polarization and promoting M2 phenotype transition. This phenotypic shift alleviates the inflammatory microenvironment and mitigates disease progression. Moreover, to further enhance tissue-specific delivery, membrane vesicles can be functionalized with targeting ligands such as hyaluronic acid or bioactive peptides, providing opportunities for synergistic targeting and expanding their therapeutic potential in complex inflammatory disorders [154,155].

Various delivery routes—including oral, rectal, intravenous, and i.p. administration—each offer distinct advantages and are suited to specific clinical scenarios, while also presenting unique technical challenges. Through the rational selection of membrane vesicle types and engineering optimization of delivery systems, these strategies are progressively overcoming the inherent limitations of conventional drug administration. Such advances have markedly enhanced the targeting specificity and therapeutic efficacy of MV-DDSs in the treatment of IBD, providing robust technological support for precise delivery and disease-specific intervention.

**Table 5:** Delivery strategies of MV-DDSs in IBD.

Delivery Route	Vesicle Types	Advantages	Limitations	Ref.
Oral	Liposomes	Patient-friendly administration Protect drugs from premature degradation High biocompatibility and loading capacity Effective delivery to intestinal tract	Susceptible to gastric acid and enzymes Low stability without protective coating Limited absorption of hydrophobic drugs	[28]
Intravenous	Liposomes	Rapid systemic distribution Active and passive targeting (e.g., EPR effect) Ability to load hydrophilic and hydrophobic drugs	Rapid clearance by RES Possible immunogenicity High production cost	[29]
Oral	Polymersomes	pH-responsive release in intestine Enhanced protection of drugs High encapsulation efficiency Controlled release at target site	Complex synthesis Potential instability during GI transit Limited clinical validation	[26]
Intravenous	EBMVs	Selective targeting of inflamed intestine Surface α4β7 integrin enables adhesion Intrinsic anti-inflammatory activity Reduction in inflammatory cytokine expression	Risk of immune reactions	[33]
Oral	PDMVs	Excellent food-derived origin High biocompatibility Stability in the gastrointestinal tract	Difficult to purify on a large scale Partial degradation by digestive enzymes remains possible Limited intestinal absorption efficiency	[32]
Oral	BMVs	Intrinsic mucosal adhesion Modulation of gut microbiota Oral convenience	Potential immunostimulation Enzyme degradation risk	[156]

## 5. Conclusions and Outlook

As summarized in the preceding sections, MV-DDSs have demonstrated remarkable promise in the treatment of IBD, by enabling targeted delivery of anti-inflammatory agents, nucleic acids, antioxidants, and biologics, while also modulating immune responses, maintaining intestinal barrier integrity, and regulating programmed cell death pathways. However, despite their compelling preclinical efficacy, the translation of these potent vesicle-based systems into clinically meaningful therapies for IBD remains elusive, largely due to unresolved technical, safety, and regulatory barriers. Addressing these issues is imperative for realizing their therapeutic potential in human patients.

1. Standardization and scalability of production. The heterogeneity of vesicle sources—including plant-derived vesicles, bacterial outer membrane vesicles, milk-derived vesicles, and engineered biomimetic vesicles—poses difficulties in achieving reproducible large-scale production with consistent physicochemical properties and bioactivity. Future efforts should focus on establishing robust manufacturing processes and standardized quality control protocols.
2. Biosafety and immunogenicity. Despite their generally favorable biocompatibility, the residual immunogenic components in certain vesicles, such as endotoxins in bacterial vesicles or allergenic proteins in milk-derived vesicles, remain a safety concern. Rigorous purification, detoxification, and characterization strategies are needed to ensure low immunogenicity without compromising therapeutic efficacy.
3. Precise targeting and controlled release. Although vesicles exhibit inherent tropism to inflamed intestinal tissues, strategies to further improve site-specific delivery and stimuli-responsive release—such as engineering vesicles to respond to local oxidative stress or pH gradients—require continued optimization.
4. Cargo loading efficiency and stability. Efficient encapsulation and stable retention of diverse therapeutic cargos, especially sensitive biological molecules like siRNA, peptides, or probiotics, remain technical bottlenecks. The development of advanced loading techniques and stabilizing formulations will be pivotal to maximize their therapeutic potential.
5. In vivo tracking and pharmacokinetics. The complex intestinal microenvironment and dynamic mucus barrier present obstacles for vesicle trafficking and persistence in vivo. New imaging modalities and tracing methods are necessary to elucidate vesicles’ biodistribution, clearance, and mechanisms of action in real time.
6. Regulatory pathways and translational readiness. Given the novelty and complexity of MV-DDSs, regulatory frameworks governing their safety, efficacy, and manufacturing standards are still evolving. Close collaboration among academic researchers, industry partners, and regulatory agencies will be essential to accelerate their clinical development.

In light of these challenges, targeted measures such as establishing unified manufacturing standards, integrating advanced bioengineering for precision delivery, and implementing rigorous preclinical-to-clinical validation pipelines will be key to bridging the gap between laboratory success and patient benefit. Overall, MVs-DDSs represent a versatile and innovative therapeutic platform with the potential to reshape IBD management. Future interdisciplinary research—uniting material science, microbiology, immunology, and clinical medicine—is expected to overcome the current barriers, enabling the development of safe, precise, and effective next-generation MV-DDSs for inflammatory bowel disease.

## Figures and Tables

**Table 1:** Related information on different MVs.

Category	Source	Size (nm)	Markers	Biogenesis	Features	Refs.
MDMVs	Mammalian cells	30–150	CD9, CD63, CD81	Endosomal budding to form multivesicular bodies, followed by membrane fusion	high biocompatibility; low immunogenicity; intrinsic targeting;	[21,22]
BMVs	Bacteria (Gram-negative, Gram-positive)	10–400	Gram-negative: LPS, OmpA/OmpC/OmpF; Gram-positive: LTA, Peptidoglycan	Outward budding from bacterial membrane	strong immune stimulation; vaccine adjuvant potential	[23,24]
PDNVs	Plants (fruits, vegetables, herbs, etc.)	50–500	PM H^+^-ATPase, PATL1	Plasma membrane invagination and exocytosis	excellent biocompatibility; specific targeting; acid-resistant;	[25]
Liposomes	Phospholipids, cholesterol	50–200	DSPC, PEG-DSPE	Self-assembly in aqueous solution	high drug loading; immune evasion; high stability; controlled release; combines natural biofunctionality with tunable synthetic properties	[26,27,28]
Polymersomes	synthetic amphiphilic block copolymers	70–300	PEG-PLA	Self-assembly in aqueous solution		[29,30,31]
EBMVs	Artificial assembly (cell membrane + synthetic materials)	20–300	Related to molecular signatures of parent cell membranes	Coating nanoparticle cores with natural or hybrid membrane		[32,33]

## Abbreviations

The following abbreviations are used in this manuscript:

IBD	Inflammatory bowel disease
CD	Crohn’s disease
UC	Ulcerative colitis
MV-DDSs	Membrane vesicle-based drug delivery systems
MDMVs	Mammalian-derived membrane vesicles
PDMVs	Plant-derived membrane vesicles
BMVs	Bacterial membrane vesicles
EBMVs	Engineered biomimetic membrane vesicles
DDSs	Drug delivery systems
MVs	Membrane vesicles
OMVs	Outer membrane vesicles
ROS	Reactive oxygen species
5-ASA	5-aminosalicylic acid
MSCs	Mesenchymal stem cells
EcN	*Escherichia coli* Nissle
EPR	Enhanced permeability and retention
PEG	Polyethylene glycol
PAMPs	Pathogen-associated molecular patterns
PDE4	Phosphodiesterase 4
Nrf2	Nuclear factor erythroid 2-related factor 2
ACNs	Anthocyanins
FU	Fucoidan
MPO	Myeloperoxidase
CeO_2_	Cerium oxide
hAFSCs	human amniotic fluid stem cells
PCD	Programmed cell death
Se	Selenium
LbL	Layer-by-layer
HTCC	N-(2-hydroxy)propyl-3-trimethylammonium chitosan chloride
OKGM	Oxidized konjac glucomannan
DMA	Dopamine methacrylamide
i.p.	Intraperitoneal
CCR2	cc chemokine receptor 2
MCP-1	Monocyte chemoattractant protein-1
